# Acute Alcohol‐Induced Changes Measured With Metabotropic Glutamate Receptor 5 Positron Emission Tomography

**DOI:** 10.1111/adb.70031

**Published:** 2025-05-01

**Authors:** Nakul R. Raval, Kelly Smart, Gustavo A. Angarita, Rachel Miller, Yiyun Huang, John H. Krystal, Richard E. Carson, Kelly P. Cosgrove, Stephanie S. O'Malley, Ansel T. Hillmer

**Affiliations:** ^1^ Yale PET Center Yale University New Haven Connecticut USA; ^2^ Department of Radiology and Biomedical Imaging Yale University New Haven Connecticut USA; ^3^ Department of Psychiatry Yale University New Haven Connecticut USA; ^4^ Connecticut Mental Health Center New Haven Connecticut USA

**Keywords:** alcohol challenge, glutamate release, metabotropic glutamate type 5 receptors, positron emission tomography, social drinkers

## Abstract

**Background:**

Alcohol consumption at clinically relevant doses alters brain glutamate release. However, few techniques exist to measure these changes in humans. The metabotropic glutamate receptor 5 (mGluR5) PET radioligand [^11^C]ABP688 is sensitive to acute alcohol in rodents, possibly mediated by alcohol effects on glutamate release. This study aimed to determine the sensitivity of [^11^C]ABP688 PET to an acute alcohol challenge in humans.

**Methods:**

Eight social drinkers (25–42 years; 5 females) with a recent drinking occasion achieving a blood alcohol level (BAL) > 80 mg/dL were recruited. All participants underwent a 90‐min dynamic baseline [^11^C]ABP688 PET scan. Two weeks later (range: 7–29 days), participants completed an oral laboratory alcohol challenge over 30 min, targeting a BAL of 60 mg/dL. Immediately after the challenge, a second [^11^C]ABP688 PET scan was performed. Non‐displaceable binding potential (*BP*_ND_; indicative of mGluR5 availability) and *R*_1_ (indicative of relative blood flow) were estimated using the simplified reference tissue model with the cerebellum as the reference region. Blood samples were taken throughout the scanning procedure to measure the BAL.

**Results:**

Seven participants (4 females) completed the study. The mean peak BAL achieved was 61 ± 18 mg/dL. Acute alcohol significantly decreased [^11^C]ABP688 *BP*_ND_, *F*(1, 42) = 17.05, *p* < 0.001, Cohen's *d* = 0.32–0.60, and increased [^11^C]ABP688 *R*_1_, *F*(1, 42) = 6.67, *p* = 0.013, Cohen's *d* = 0.32–0.48, across brain regions. Exploratory analysis showed a positive relationship between alcohol‐induced % change in [^11^C]ABP688 *R*_1_ in cortical regions and peak BAL (Spearman rho = 0.78 [frontal cortex] and 0.85 [temporal cortex] = 0.024 and 0.011).

**Conclusions:**

This proof‐of‐concept study demonstrates that [^11^C]ABP688 PET imaging is sensitive to the effects of acute alcohol consumption. The observed decrease in mGluR5 availability aligns with preclinical data potentially indicating acute increased extracellular glutamate concentrations following ethanol dosing. This imaging tool could be useful for future investigations into the acute effects of alcohol on the brain during abstinence and withdrawal.

## Graphical abstract

This proof‐of‐concept study provides evidence that acute alcohol administration reduces mGluR5 availability and increases relative blood flow in the human brain, as measured by [11C]ABP688 PET. The observed effects align with preclinical evidence of alcohol‐induced glutamate release. These findings support the utility of [11C]ABP688 PET for probing glutamatergic changes during alcohol exposure in humans.

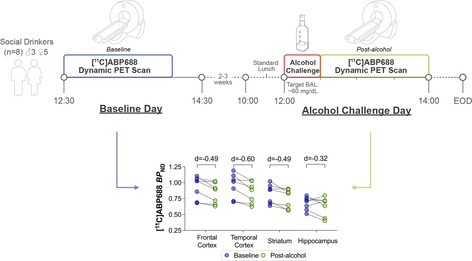

## 1 Introduction

Glutamate is the primary excitatory neurotransmitter in the mammalian brain, playing a central role in physiological processes such as learning, memory, and neuroplasticity. Dysregulated glutamate signalling is reported with both acute and chronic alcohol exposure [1]. Ethanol has dose‐related effects on extracellular limbic glutamate levels in rats, with doses associated with social drinking (0.5 g/kg) elevating extracellular glutamate levels, while much higher levels (2.0 g/kg) reduce these levels [2]. In models of binge‐like consumption [3, 4, 5], alcohol initially raises extracellular glutamate levels, but then glutamate release levels drop below baseline as blood alcohol levels (BAL) decline [2, 6, 7]. Chronic alcohol exposure has been associated with excessive extracellular glutamate levels and excitatory signalling [7, 8, 9, 10]. Importantly, disrupted glutamate signalling has been associated with craving and relapse propensity during abstinence [11]. While tools to measure glutamate release in rodents are available, there are limited tools to measure changes in glutamate release in people [12].

Studies have shown that positron emission tomography (PET) imaging with the radiotracer [^11^C]ABP688 [3‐((6‐methylpyridin‐2‐yl)ethynyl) cyclohex‐2‐en‐1‐one‐O‐[^11^C]methyloxime] is sensitive to changes in extracellular glutamate levels. [^11^C]ABP688 binds selectively to the allosteric site of metabotropic glutamate receptor subtype 5 (mGluR5) [13, 14], which are excitatory Gq‐coupled G protein receptors predominantly expressed on the postsynaptic sites of neurons [15]. Acute administration of ketamine, which transiently elevates glutamate levels, reduces [^11^C]ABP688 volume of distribution (*V*_T_) [16, 17], supporting the use of [^11^C]ABP688 PET to measure changes in extracellular glutamate concentrations. A recent rodent study used simultaneous microdialysis and [^11^C]ABP688 microPET imaging to show concurrent increases in glutamate release and decreases in striatal [^11^C]ABP688 binding following administration of ethanol [18]. This is consistent with prior reports of calcium‐dependent glutamate release in the rodent brain following ethanol administration [2, 6, 7, 19]. Motivated by these findings, the goal of this study was to translate these findings to people by evaluating the mGluR5 response to an acute laboratory alcohol challenge with [^11^C]ABP688 and PET. The hypothesis for this study was that acute alcohol consumption would decrease mGluR5 availability in the brain. A secondary goal was to evaluate the acute alcohol effects on relative [^11^C]ABP688 delivery (*R*_1_), a surrogate of relative blood flow with other radiotracers [20, 21], as acute alcohol is known to increase blood flow [22, 23, 24].

## 2 Material and Methods

### 2.1 Recruitment and Study Participants

The Yale School of Medicine Human Investigation Committee and the Radiation Safety Committee approved all procedures. Study participants were recruited from the local New Haven population. Participants self‐reported at least a single drinking occasion sufficient to reach an estimated BAL of 80 mg/dL in the past 3 months, operationally defined as more than three drinks for females and more than four drinks for males at intake. This ensured that study participants had prior drinking experience consistent with levels achieved in this study. Participants were asked to recall the two heaviest days of drinking in the previous 3 months, useful for calculating the BAL achieved for those episodes.

Prior to their participation, all subjects provided written informed consent. Recruited participants had no current or past significant medical or neurological disorders and did not meet DSM‐5 criteria for current or past psychiatric or substance use disorders. Subjects who had a history of perceptual distortions, seizures, delirium, or hallucinations upon alcohol withdrawal or scored greater than 12 on the Clinical Institute Withdrawal Assessment scale at intake appointments were excluded. Additionally, participants did not use psychotropic medication over the month prior to participation. Participants for whom alcohol consumption was medically contraindicated were also excluded. Negative pregnancy tests were required for all females during screening and on the day of radiotracer administration. During intake and on scan day, alcohol consumption over the prior 30 days was recorded with the Alcohol Timeline Followback Interview [24]. A total of eight social drinkers (five women and three men) were recruited to participate (see Section 3 and Table 1 for demographics). One subject met the criteria for mild AUD.

**TABLE 1:** Participant demographics and scanning parameters.

Age	27.7 ± 6 years (range: 22–42)
Sex	3M; 4F
Number of drinks in the last 30 days on scan day	22 ± 20 drinks (range: 2–53)
AUD status (DSM‐5)	1 mild AUD (1F)
Peak BAL	61 ± 18 mg/dL (*n* = 7)
Days between scan	17 ± 8 days (range: 7–29)
Injected dose	Baseline: 614.5 ± 62 MBq (range: 530–684)
	Post‐alcohol: 638 ± 53 MBq (range: 529–688)
Injected mass (E)‐isomer only	Baseline: 0.36 ± 0.1 μg (range: 0.19–0.43)
	Post‐alcohol: 0.39 ± 0.1 μg (range: 0.27–0.54)
[^11^C]ABP688% (E)‐isomer	Baseline: 70 ± 10 (range: 58–83)
	Post‐alcohol: 70 ± 6 (range: 63–81)
Scan start time	Baseline: 12:17 (range: 11:55–12:35)
	Post‐alcohol: 12:24 (range: 11:59–12:36)

*Note:* No significant differences were noted in the [^11^C]ABP688 injected dose or mass.

Abbreviations: AUD, alcohol use disorder; F, female; M, male.

### 2.2 Experimental Design

**FIGURE 1:**
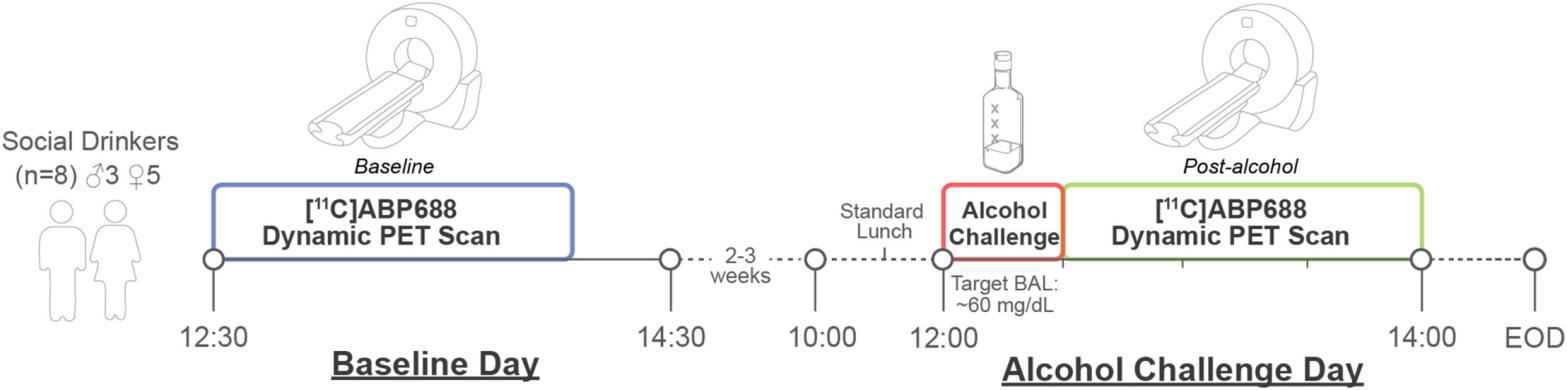
Study design. Eight social drinkers had a baseline 90‐min [^11^C]ABP688 PET scan. Two to three weeks later, they completed a 30‐min alcohol session targeting BAL ~60 mg/dL, followed by a second 90‐min PET scan. Scans were done at the same time of day to control for diurnal variations.

All subjects participated in two [^11^C]ABP688 PET scans and a laboratory alcohol drinking session (see Figure 1). Participants were asked to abstain from alcohol for at least 48 h prior to the study day, confirmed by self‐report. Abstinence on the morning of scanning was confirmed with a negative breath alcohol test. Baseline [^11^C]ABP688 PET scans were acquired on the ‘Baseline Day’. Two to three weeks after the Baseline Day, participants came in for the ‘Alcohol Challenge Day’. The Alcohol Challenge Day started with a standardized lunch. Next, at approximately 12:00 pm, participants consumed an alcohol dose calculated to achieve a BAL of at least 60 mg/dL. The dose was prepared taking into account the participant's total body water (based on sex, age, height, and weight), duration of drinking and ratio of alcohol to mixer, based the Widmark equation as updated by Watson et al. [25]. Alcohol was administered as 80‐proof vodka mixed with a decarbonated, non‐caffeinated, and non‐caloric drink of the participant's choice at a 1:3 alcohol‐to‐mixer ratio. The total volume was divided into three equal drinks, with each consumed over a 10‐min period to pace the rate of consumption, with the total duration of drinking being 30 minutes. Immediately after the completion of the laboratory alcohol session, the post‐alcohol [^11^C]ABP688 PET scan was acquired. To avoid the diurnal effects of [^11^C]ABP688 [26], PET scans were scheduled at the same time on different days (approximately 12:30 pm).

The Biphasic Alcohol Effects Scale (BAES) [27] and Drug Effects Questionnaire (DEQ) [28] were used to assess the subjective effects of alcohol. BAES is a 14‐item questionnaire on an 11‐point scale measuring alcohol's stimulating and sedating effects. DEQ, a 5‐item visual analogue scale, evaluates the subjective effects of alcohol and includes items assessing ‘FEEL’ and ‘HIGH’ drug effect. BAES was measured at baseline (roughly 5 min prior to the start of the alcohol session), and every 30 min for at least 180 min after the start of the alcohol session. DEQ was measured at baseline and every 15 min until at least 45 min after the start of the alcohol session.

To measure BAL, venous blood samples were acquired at 30‐min intervals from the start of the alcohol drinking session until the end of the scanning routine, including a baseline sample approximately 5 min before the start of the session. BAL was measured with headspace gas chromatography at the Yale‐New Haven Hospital Clinical Laboratories using their standard protocol.

### 2.3 Imaging Data Acquisition

[^11^C]ABP688 of high E/Z ratio (70:1) [16] was synthesized at the Yale PET Center as previously described [29], resulting in high molar activities of 420 ± 129 GBq/μmol (minimum = 299 GBq/μmol). PET data were acquired with a High Resolution Research Tomograph (Siemens Medical Solutions USA Inc., Malvern, PA, USA). Head motion data were acquired with an optical motion‐tracking tool (Vicra; NDI Systems, Waterloo, ON, Canada). A 6‐min transmission scan was acquired for attenuation correction prior to the radiotracer injection. PET data acquisition began simultaneously with the administration of [^11^C]ABP688 as a slow bolus over 1 min. Dynamic PET data were acquired for 90 min alongside arterial blood sampling to measure the metabolite‐corrected input function [16].

On a separate day, all participants underwent T1‐weighted structural magnetic resonance (MR) scans, acquired with a Siemens 3.0T scanner (Siemens Medical Solutions USA Inc., Malvern, PA, USA) equipped with a 64‐channel head coil, providing high‐resolution anatomical maps for PET data coregistration. A sagittal gradient‐echo MPRAGE sequence was employed (FOV: 256 × 256 mm^2^, 176 slices at 1 mm thickness, TE: 2.77 ms, TR: 2530 ms, TI: 1100 ms, FA: 7°).

### 2.4 Preprocessing and Kinetic Modeling

Dynamic list‐mode brain PET data were binned into discrete time frames of increasing length up to 5 min and reconstructed with the MOLAR algorithm [30]. The first 10 min of PET brain data were registered to the subject‐specific T1‐weighted MRI using a mutual information algorithm with six degrees of freedom (FLIRT, FSL 3.2; Analysis Group; FMRIB, Oxford, UK). To define the regions of interest, the native MRI was co‐registered to the Montreal Neurological Institute template space with a nonlinear transformation algorithm (BioImage Suite; http://www.bioimagesuite.com). Time–activity curves were generated from the frontal cortex, temporal cortex, striatum, hippocampus, and cerebellum from the Automated Anatomical Labelling (AAL) Atlas [31]. These regions were chosen due to their known involvement in glutamate neurotransmission and their relevance to the neurobiological effects of alcohol. The whole striatum was analysed to account for PET resolution limitations and to reduce variability in this exploratory study. Regions of interest were grey matter masked as assessed by CAT (A Computational Anatomy Toolbox for Statistical Parametric Mapping [SPM12; Institute of Neurology, University College of London, London, England]; Jena University Hospital, Jena, Germany).

For a subset of the participants (*n* = 4, 2 males, 2 females), arterial blood samples were collected throughout both the scanning procedures. [^11^C]ABP688 volumes of distribution (*V*_T_) were calculated in the selected regions, including the cerebellum. *V*_T_ is the ratio of [^11^C]ABP688 concentration in tissue to [^11^C]ABP688 concentration in arterial plasma at equilibrium and was estimated with the two tissue compartment model (2TCM). However, arterial blood sampling was not available for both scans in three participants, leading us to primarily use [^11^C]ABP688 non‐displaceable binding potential (*BP*_ND_) as the outcome measure for this study. *BP*_ND_ provides a measure of receptor availability in the brain regions, indicating the density of available receptors in relation to non‐displaceable binding.

*BP*_ND_ and *R*_1_ were estimated using the simplified reference tissue model [32] with the cerebellum as the reference region. While the cerebellum is commonly used as a reference region due to its minimal specific binding [33, 34], there is evidence for small amounts of mGluR5‐specific binding, which could bias *BP*_ND_ estimates (see Section 4). *R*_1_ values, defined as the ratio of rate constants (*K*_1_) describing tracer influx from plasma to the target region and to the reference region, were also estimated. The *R*_1_ value quantifies relative radiotracer delivery to different brain regions. Since [^11^C]ABP688 has high first‐pass extraction [35], *R*_1_ provides a proxy measure of relative blood flow as a complement to *BP*_ND_ analyses, providing a more comprehensive understanding of the acute effects of alcohol on brain function.

### 2.5 Statistical Analysis

Separate linear mixed‐effects models were employed for the statistical analysis of [^11^C]ABP688 *BP*_ND_ and *R*_1_, following confirmation of the data's normal distribution. The constructed model incorporated *PET state* (baseline vs. post‐alcohol) and *Region* as fixed effects, along with their interaction, while a random intercept accounted for individual variability (subject ID). Post hoc pairwise comparisons (Fisher's least significant difference) were utilized to determine the impact of alcohol on the frontal cortex, temporal cortex, hippocampus and striatum.

Alcohol‐induced Δ*BP*_ND_ and Δ*R*_1_ were quantified as percentage differences ([Post‐alcohol − Baseline]/Baseline * 100). Exploratory analyses examined potential relationships between Δ*BP*_ND_ and Δ*R*_1_ with the following factors: (1) subjective alcohol effects, (2) peak BAL and (3) self‐reported alcohol consumption over the past month. These analyses were designed to test hypotheses concerning the mGluR5 response to alcohol: (1) its association with subjective alcohol responses, (2) its dependence on alcohol dosage and (3) its correlation with recent drinking history. Subjective effects analyses focused on stimulation during the ascending limb (30‐min intervals of BAES stimulation, DEQ FEEL and DEQ HIGH) and sedation during the descending limb (150‐ and 210‐min intervals of BAES sedation). Further partial correlation analyses, controlling for baseline subjective effects, were conducted to explore the associations between Δ*BP*_ND_ and Δ*R*_1_ and BAL measures, as well as recent drinking history. Given the exploratory nature of these analyses, Spearman rank correlation coefficients (Spearman's rho) were calculated without correction for multiple comparisons.

Scan characteristics and *V*_T_ value comparisons were made using paired *t*‐tests. Statistical analyses were performed using R 4.2.2 (‘*Innocent and Trusting*’) and RStudio (RStudio Team, Boston, MA, USA), with data visualization carried out using GraphPad Prism (v. 9.4.1; GraphPad Software, San Diego, CA, USA).

## 3 Results

### 3.1 Participant Demographics and Characteristics

**FIGURE 2:**
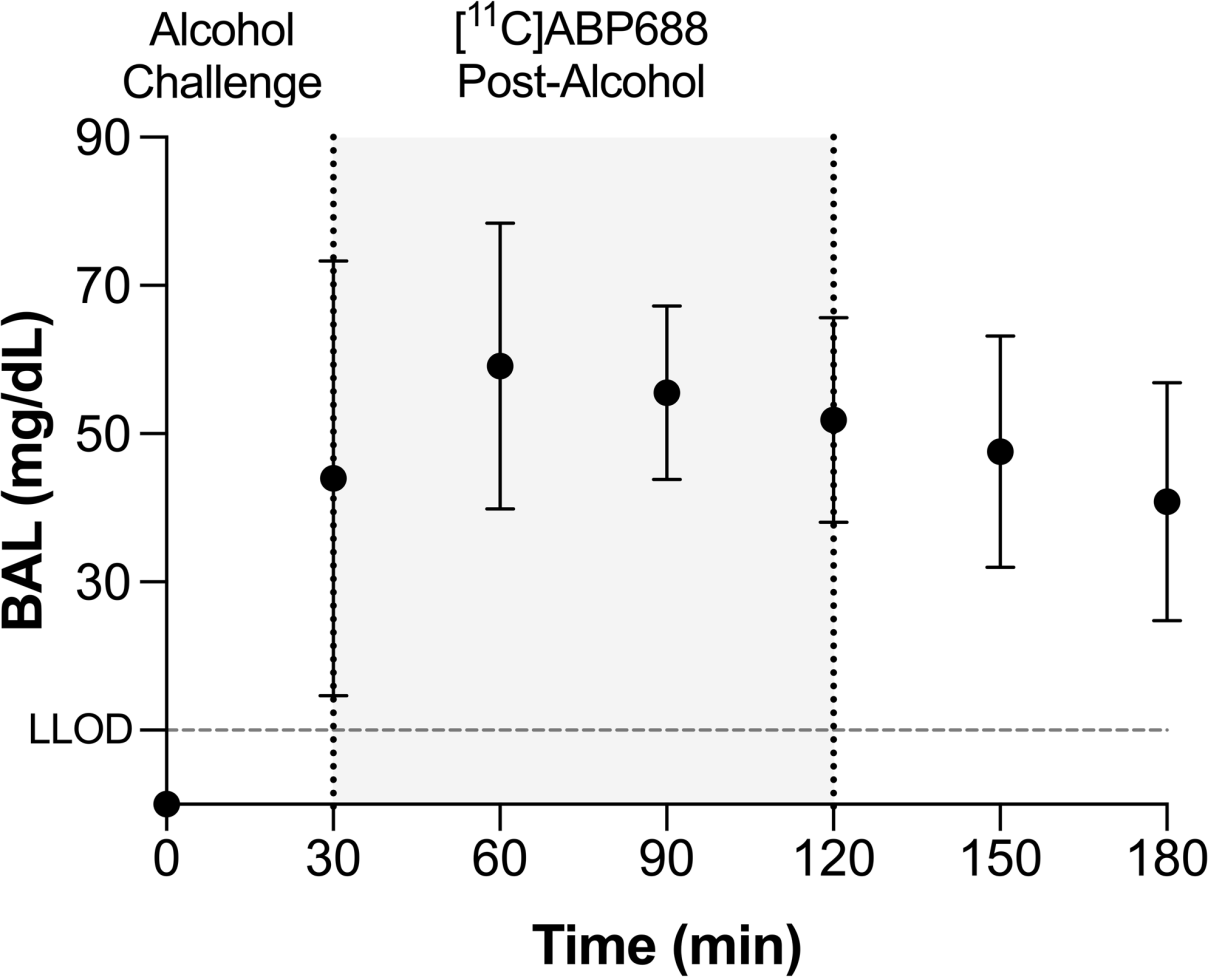
Mean blood alcohol level (BAL, *n* = 7) during and after the alcohol drinking session lasting 30 min. Error bars denote the standard deviation. Horizontal dashed line denotes the lower limit of detection (LLOD).

Eight participants (3 males, 5 females) enrolled in the study. One female met DSM‐5 criteria for mild alcohol use disorder. All participants reported being non‐smokers. One participant did not complete the study, vomited during the laboratory alcohol session, with a peak BAL of 33 mg/dL at 120 min. This participant was excluded from the final analysis. The final analysis included seven participants (3 males, 4 females) with an average age of 27.7 ± 6 years. These participants reported consuming an average of 22 ± 20 drinks in the past 30 days. During the laboratory drinking session, males consumed 150.7 ± 11 mL of 80‐proof alcohol, while females consumed 111.6 ± 20 mL. The average peak BAL among the included participants was 61.4 ± 18 mg/dL, exhibiting a typical biphasic BAL curve (see Figure 2). Behavioural data indicated that alcohol consumption led to a decrease in stimulation and an increase in sedation, as measured by the BAES, and in subjective feelings of intoxication, as measured by FEEL and HIGH on the DEQ (Figure S1). For PET scans, no significant differences were observed in injected activity, injected mass, [^11^C]ABP688 E/Z ratio, or the time of scan between the baseline and post‐alcohol scans (Table 1).

### 3.2 Using the Cerebellum as a Reference Region

Analysis of [^11^C]ABP688 *V*_T_ was restricted to the four participants for whom arterial input function was acquired for both scans (see Tables S1 and S2). Alcohol did not significantly alter the [^11^C]ABP688 parent fraction (60–90 min post‐injection: 12 ± 5% at baseline; 13 ± 6% post‐alcohol) or arterial input function (SUV 60–90 min post‐injection: 0.12 ± 0.05% at baseline; 0.12 ± 0.07 post‐alcohol). The cerebellum *V*_T_ exhibited little change from baseline (1.95 ± 0.4; range = 1.66–2.44) compared with post‐alcohol (2.03 ± 0.3; range = 1.71–2.46), with an overall change of 4.5 ± 4% (range = 0.07–8.58%), within the reported cerebellum [^11^C]ABP688 *V*_T_ test–retest variability of 13% [27]. This result was taken to support the use of the cerebellum as a reference region for this study design.

### 3.3 Acute Alcohol Decreases Brain mGluR5 Availability

**FIGURE 3:**
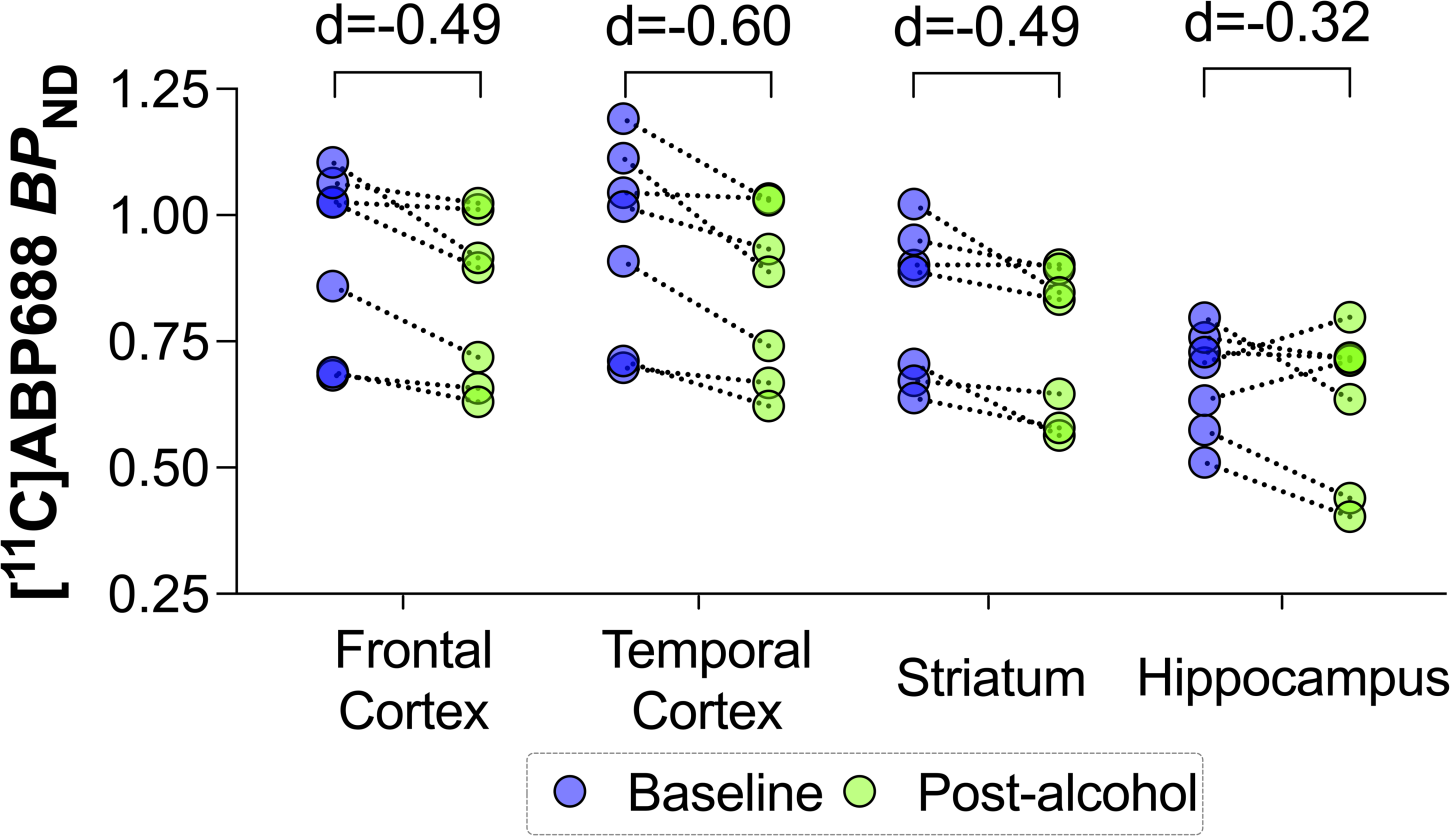
[^11^C]ABP688 *BP*_ND_, indicative of mGluR5 availability, decreases in all regions after the laboratory alcohol challenge (*n* = 7). Cohen's *d* values are presented.

Brain mGluR5 availability, quantified by [^11^C]ABP688 *BP*_ND_, significantly decreased post‐alcohol compared to baseline. The main effect of alcohol, *F*(1, 42) = 17.05, *p* < 0.001, was statistically significant. The interaction between alcohol and region was not statistically significant, *F*(4, 42) = 0.57, *p* = 0.632, suggesting that the decrease in mGluR5 availability was potentially a whole‐brain effect. On average, [^11^C]ABP688 *BP*_ND_ values decreased by 9% post‐alcohol compared to baseline, as illustrated in Figure 3. Post hoc analysis revealed moderate effect sizes of alcohol in the following brain regions: frontal cortex (9.08% decrease, Cohen's *d* = 0.49, *p* = 0.015), temporal cortex (11.2% decrease, Cohen's *d* = −0.60, *p* = 0.010), striatum (8.9% decrease, Cohen's *d* = −0.49, *p* = 0.021) and hippocampus (6.6% decrease, Cohen's *d* = −0.32, *p* = 0.317).

**FIGURE 4:**
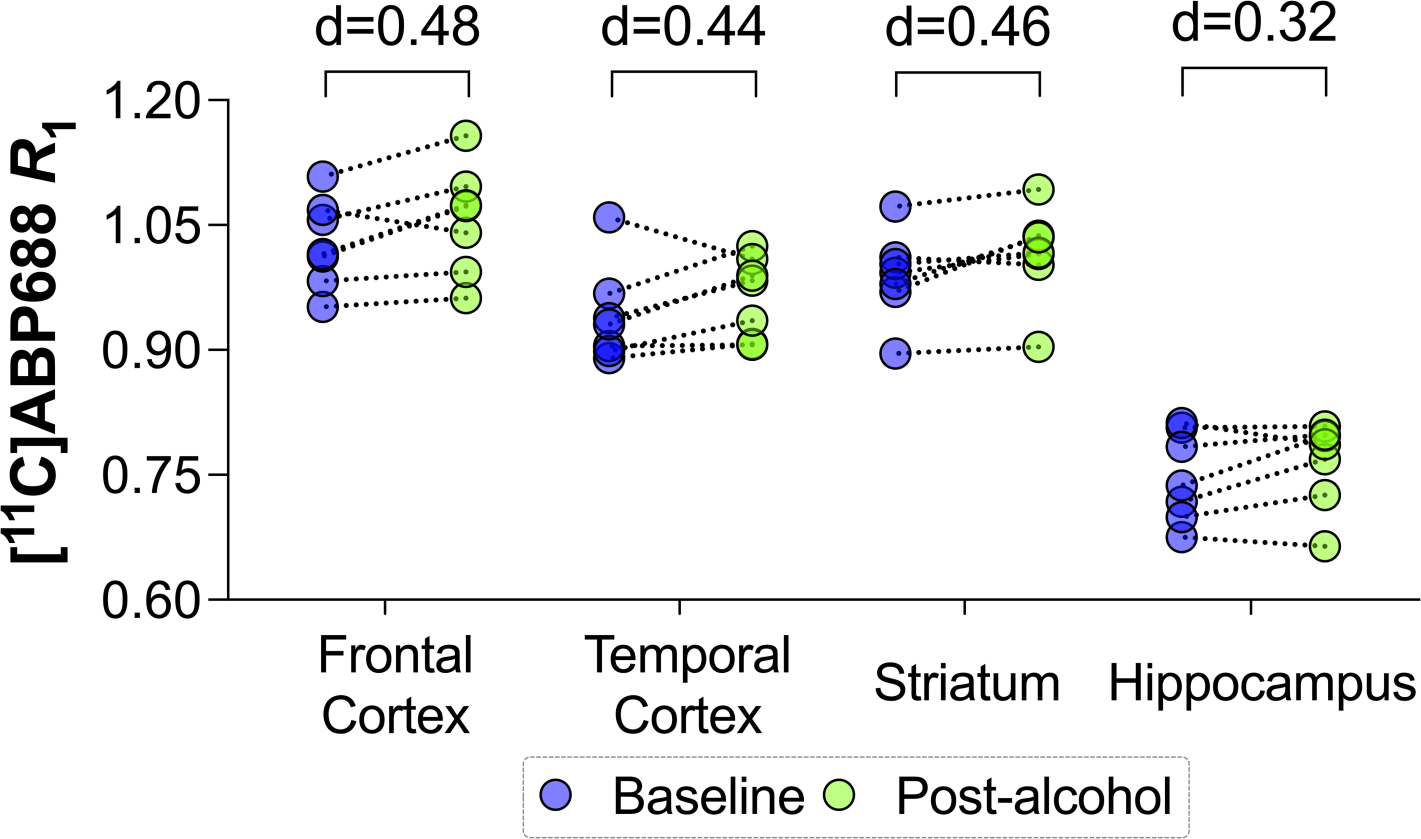
[^11^C]ABP688 *R*_1_, indicative of relative blood flow, increased in most regions after the laboratory alcohol challenge *(n* = 7). Cohen's *d* values are presented.

Relative blood flow, quantified by [^11^C]ABP688 *R*_1_, significantly increased post‐alcohol compared to baseline, as illustrated in Figure 4. The main effect of alcohol, *F*(1, 42) = 6.69, *p* = 0.013, was statistically significant, while the interaction between alcohol and region was not statistically significant, *F*(4, 42) = 0.08, *p* = 0.970. Exploratory post hoc analysis revealed a significant increase in the striatum (2.59% increase, Cohen's *d* = 0.46, *p* = 0.046) but no significant changes in the frontal cortex, hippocampus or temporal cortex (see Figure 4).

### 3.4 Relationship of [^11^C]ABP688 mGluR5 Availability and Relative Blood Flow With Peak BAL, Recent Drinking Behaviour and Subjective Effects

**FIGURE 5:**
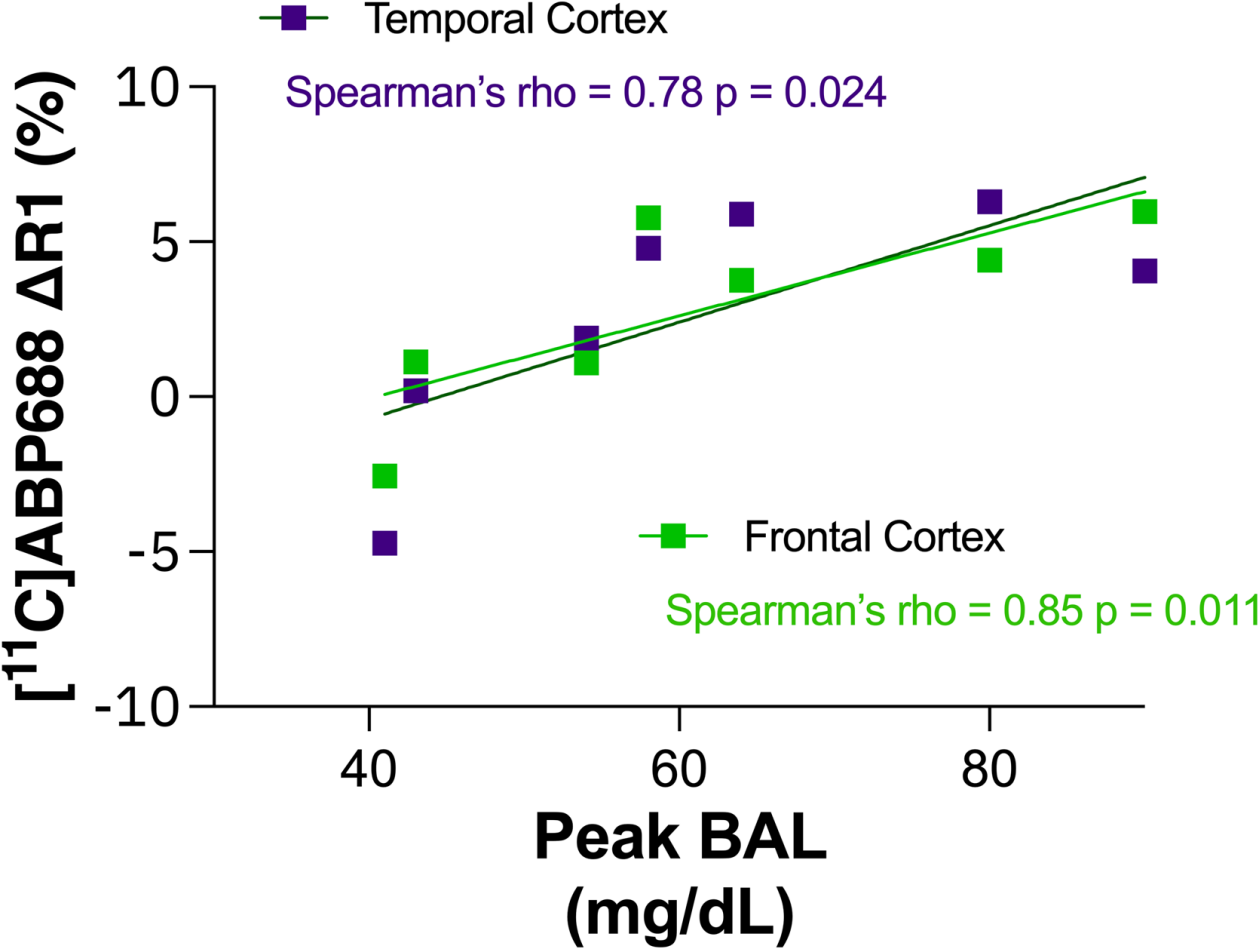
Relationships between [^11^C]ABP688 Δ*R*_1_, indicative of percent change in relative blood flow after alcohol, and peak BAL. Spearman's rho is colour‐coded for the frontal and temporal cortices and inserted in the figure.

Exploratory analyses revealed no significant relationships between Δ*BP*_ND_ and peak BAL, number of drinks in the past month or subjective effects such as BAES or DEQ. In contrast, there was initial evidence for significant (uncorrected for multiple comparisons) positive relationships between Δ*R*_1_ and peak BAL in frontal cortex (Spearman rho = 0.85; *p* = 0.011) and temporal cortex (Spearman rho = 0.78; *p* = 0.024), as illustrated in Figure 5. No other significant relationships were found between Δ*R*_1_ and peak BAL, recent drinking amounts or subjective effects like BAES or DEQ (Tables S4 and S5).

## 4 Discussion

This study reveals evidence that a laboratory alcohol challenge achieving an average peak BAL of ~60 mg/dL significantly reduced brain mGluR5 availability, as measured by [^11^C]ABP688 *BP*_ND_. Further analyses yielded modest evidence that alcohol increased relative blood flow in cortical regions, as measured by [^11^C]ABP688 *R*_1_, with positive correlations between *R*_1_ increases and peak BAL, largely confirming prior findings (see below). No significant associations were found between post‐alcohol Δ*BP*_ND_ or Δ*R*_1_ and recent drinking behaviour or subjective effects. The findings establish a novel imaging paradigm for investigating the dynamic effects of acute alcohol on brain glutamate function.

This in vivo human evidence for imaging [^11^C]ABP688 response to alcohol aligns with preclinical rodent literature [35], suggesting that alcohol‐induced glutamate release contributes to the observed decrease in mGluR5 availability. Despite the preliminary nature of the data reported here, it is noteworthy that all subjects exhibited a consistent decrease in [^11^C]ABP688 binding. There are mixed preclinical findings regarding the sensitivity of [^11^C]ABP688 PET imaging to extracellular glutamate, as pharmacological challenges designed to alter glutamate levels in rodents yielded no observable effect [36, 37], while nonhuman primate studies provide evidence, albeit with caveats, supporting this mechanism [29, 38]. However, the largest dataset available in the literature is a human study reporting [^11^C]ABP688 sensitivity to ketamine [16], and this sensitivity was reproduced in an independent human cohort with a different PET radiotracer targeting mGluR5 [39]. Combined with recently reported simultaneous microdialysis/PET results [18], the presented data align with these latter reports, supporting the use of [^11^C]ABP688 PET imaging to measure alcohol‐induced changes in mGluR5 availability, although the results should be validated in larger cohorts.

A potential mechanism for a decrease in [^11^C]ABP688 binding is that glutamate causes mGluR5s to internalize, reducing their availability for [^11^C]ABP688 binding since this radiotracer targets an allosteric site on cell surface receptors only [40]. Microdialysis results in rodents suggest that alcohol doses of 0.5 g/kg increase extracellular glutamate by 200%, while larger alcohol doses elicit weaker effects [19]. mGluR5 internalization has been observed at both high and low agonist concentrations and can occur independently of agonist binding [41, 42]. However, recent findings failed to demonstrate a significant correlation between changes in [^11^C]ABP688 *BP*_ND_ and glutamate release, although anaesthesia effects may have interfered with this relationship [18], and alternative orthosteric mechanisms affecting [^11^C]ABP688 binding cannot be ruled out. The timeline of these fluctuations is also critical for interpreting receptor availability changes. Nonetheless, the observed decrease in [^11^C]ABP688 *BP*_ND_ following acute alcohol highlights potential disruptions in glutamate signaling pathways, which are essential for synaptic plasticity and cognitive functions [43]. In addition, mGluR5 receptors on astrocytes are key to astrocytic Ca(2+) signalling and neuron–glia interactions and mediate alcohol‐induced neurotoxicity, including disruptions in glutamate homeostasis. Such disruptions can lead to long‐term changes in glutamate function and behaviour associated with AUD and subsequent recovery [11, 44] and are important areas of research for future studies that may leverage this imaging paradigm to evaluate targeted therapies aimed at modulating glutamate signalling.

The primary outcome measure for this study was [^11^C]ABP688 *BP*_ND_ using the cerebellum as a reference region. This allowed for the use of the full dataset, as arterial blood sampling was available for both baseline and challenge studies in only 4 participants. The cerebellum contains small but significant [^11^C]ABP688 specific binding, which could introduce systematic biases and potentially reduce the accuracy of detected associations [45, 46]. Cerebellum [^11^C]ABP688 *V*_T_ values were carefully examined in the data and no significant changes were found after the acute alcohol, supporting the use of this reference region in the context of this challenge. Further analysis of late SUV values (60–90 min post‐injection) similarly indicated no significant difference in cerebellum SUV but lower SUV in frontal cortex and temporal cortex (see Table S3). Should alcohol cause small reductions in cerebellar [^11^C]ABP688 specific binding, this would lead to underestimation of decreases in [^11^C]ABP688 BP_ND_. Detecting significant changes despite this potential underestimation strengthens the validity of this analytic approach in the context of alcohol challenge. Indeed, [^11^C]ABP688 *BP*_ND_ values derived using the cerebellum correlate well with [^11^C]ABP688 *V*_T_ obtained from arterial input herein (*r* = 0.60, *p* = 0.0002) and elsewhere [47], and the inherent variability of [^11^C]ABP688 *V*_T_ values [26, 48] is poorer than that of [^11^C]ABP688 *BP*_ND_ [33]. Further investigation into alcohol's effects on these areas will require larger sample sizes to improve statistical confidence in the findings. Moreover, complementary approaches, such as glutamate‐magnetic resonance spectroscopy, can provide additional validation and bolster confidence in these results. Taken together, this supports the use of [^11^C]ABP688 *BP*_ND_ as the primary outcome for this study, although caution must be used when interpreting study results.

Previous human imaging studies with PET [49, 50, 51, 52] and arterial spin labelling [21, 22, 23, 53, 54] have shown that alcohol stimulates cortical grey matter haemodynamics across varying BALs. In line with these findings, our study observes a modest increase in [^11^C]ABP688 *R*_1_ values, which represent relative blood flow. This increase in *R*_1_ highlights the vasodilatory effect of acute alcohol consumption. Importantly, a strong positive correlation was found between peak BAL and Δ*R*_1_, particularly in cortical grey matter regions, similar to previous studies [49, 50, 55]. These findings highlight the necessity of accounting for regional blood flow alterations when studying alcohol's impact on the brain, especially in fMRI studies of both resting state and task‐based activities, as well as in multimodal (including PET and fMRI) approaches [56].

In conclusion, this study provides in vivo human evidence that an oral alcohol challenge decreased [^11^C]ABP688 *BP*_ND_ values in cortical and subcortical regions, which may reflect glutamate fluctuations in the brain as previously reported in preclinical studies. These results establish a novel imaging paradigm that allows for the examination of the dynamic effects of acute alcohol on mGluR5 radiotracer binding in the human brain, providing a tool for future research investigating glutamatergic underpinnings of AUD.

## Author Contributions

A.T.H., S.S.O.M., K.P.C., R.E.C. and J.H.K. conceived of the initial study design. A.T.H. and R.M. obtained and maintained regulatory approvals. A.T.H., K.P.C. and R.E.C. oversaw data acquisition. N.R.R., K.S., R.M. and A.T.H. performed data analysis. G.A.A. was a study physician. Y.H. was a senior radiochemist. N.R.R. and A.T.H. prepared initial manuscript draft and figures. All authors contributed edits and revisions to the final manuscript.

## Conflicts of Interest

The authors declare no conflicts of interest.

## Supporting information


**Figure S1.** Behavioural data showing the effects of alcohol consumption on (A) stimulation and (B) sedation, measured by the Biphasic Alcohol Effects Scale (BAES), and (C) FEEL and (D) HIGH ratings, measured by the Drug Effects Questionnaire (DEQ). Data points represent mean values, and error bars indicate the standard error of the mean (SEM). The shaded areas denote the period of [^11^C]ABP688 PET imaging post‐alcohol challenge.
**Table S1**. [^11^C]ABP688 *V*_T_ before and after the alcohol challenge (*n* = 4).
**Table S2**. [^11^C]ABP688 *K*_1_ before and after the alcohol challenge (*n* = 4).
**Table S3**. [^11^C]ABP688 standardized uptake value (SUV) values before and after the alcohol challenge (*n* = 7).
**Table S4**. Partial correlation analysis between post‐alcohol Δ*BP*_ND_ values and peak blood alcohol levels (BAL), drinks in the last 30 days as measures with Alcohol Timeline Followback questionnaire, biphasic alcohol effect scale (BAES) sedation (90 min post‐alcohol) and simulation (30 min post‐alcohol), drug effect questionnaire (DEQ) FEEL and DEQ HIGH (30 min post‐alcohol) (*n* = 7).
**Table S5**. Partial correlation analysis between post‐alcohol Δ*R*_1_ values and peak blood alcohol levels (BAL), drinks in the last 30 days as measures with Alcohol Timeline Followback questionnaire, biphasic alcohol effect scale (BAES) sedation (90 min post‐alcohol) and simulation (30 min post‐alcohol), drug effect questionnaire (DEQ) FEEL and DEQ HIGH (30 min post‐alcohol) (*n* = 7).

## Data Availability

The data that support the findings of this study are available from the corresponding author upon reasonable request.
