# Supplementary material for: Acute Alcohol‐Induced Changes Measured With Metabotropic Glutamate Receptor 5 Positron Emission Tomography
Source: Addict Biol. 2025 May 1;30(5):e70031. doi: 10.1111/adb.70031 (PMC12044519; doi:10.1111/adb.70031)
Supplement: Supplementary file 1 — Figure S1. Behavioural data showing the effects of alcohol consumption on (A) stimulation and (B) sedation, measured by the Biphasic Alcohol Effects Scale (BAES), and (C) FEEL and (D) HIGH ratings, measured by the Drug Effects Questionnaire (DEQ). Data points represent mean values, and error bars indicate the standard error of the mean (SEM). The shaded areas denote the period of [11C]ABP688 PET imaging post‐alcohol challenge. Table S1. [11C]ABP688 V T before and after the alcohol challenge (n = 4). Table S2. [11C]ABP688 K 1 before and after the alcohol challenge (n = 4). Table S3. [11C]ABP688 standardized uptake value (SUV) values before and after the alcohol challenge (n = 7). Table S4. Partial correlation analysis between post‐alcohol ΔBP ND values and peak blood alcohol levels (BAL), drinks in the last 30 days as measures with Alcohol Timeline Followback questionnaire, biphasic alcohol effect scale (BAES) sedation (90 min post‐alcohol) and simulation (30 min post‐alcohol), drug effect questionnaire (DEQ) FEEL and DEQ HIGH (30 min post‐alcohol) (n = 7). Table S5. Partial correlation analysis between post‐alcohol ΔR 1 values and peak blood alcohol levels (BAL), drinks in the last 30 days as measures with Alcohol Timeline Followback questionnaire, biphasic alcohol effect scale (BAES) sedation (90 min post‐alcohol) and simulation (30 min post‐alcohol), drug effect questionnaire (DEQ) FEEL and DEQ HIGH (30 min post‐alcohol) (n = 7). [file ADB-30-e70031-s001.docx]

# Supplementary Information

# Title

Acute Alcohol-Induced Glutamate Changes Measured with Metabotropic Glutamate Receptor 5 Positron Emission Tomography

# Authors

Nakul R. Raval^1,2^, Kelly Smart^1,2^, Gustavo A. Angarita^3,4^, Rachel Miller^1^, Yiyun Huang^1,2^, John H. Krystal^3^, Richard E. Carson^1,2^, Kelly P. Cosgrove^2,3^, Stephanie S. O’Malley^3^, Ansel T. Hillmer^1,2,3^

^1.^Yale PET Center, Yale University, New Haven, CT, USA.

^2.^Department of Radiology and Biomedical Imaging, Yale University, New Haven, CT, USA.

^3.^Department of Psychiatry, Yale University, New Haven, CT, USA.

^4.^Connecticut Mental Health Center, New Haven, Connecticut, USA

For correspondence or reprints contact:

Ansel T. Hillmer

[ansel.hillmer@yale.edu](mailto:ansel.hillmer@yale.edu)


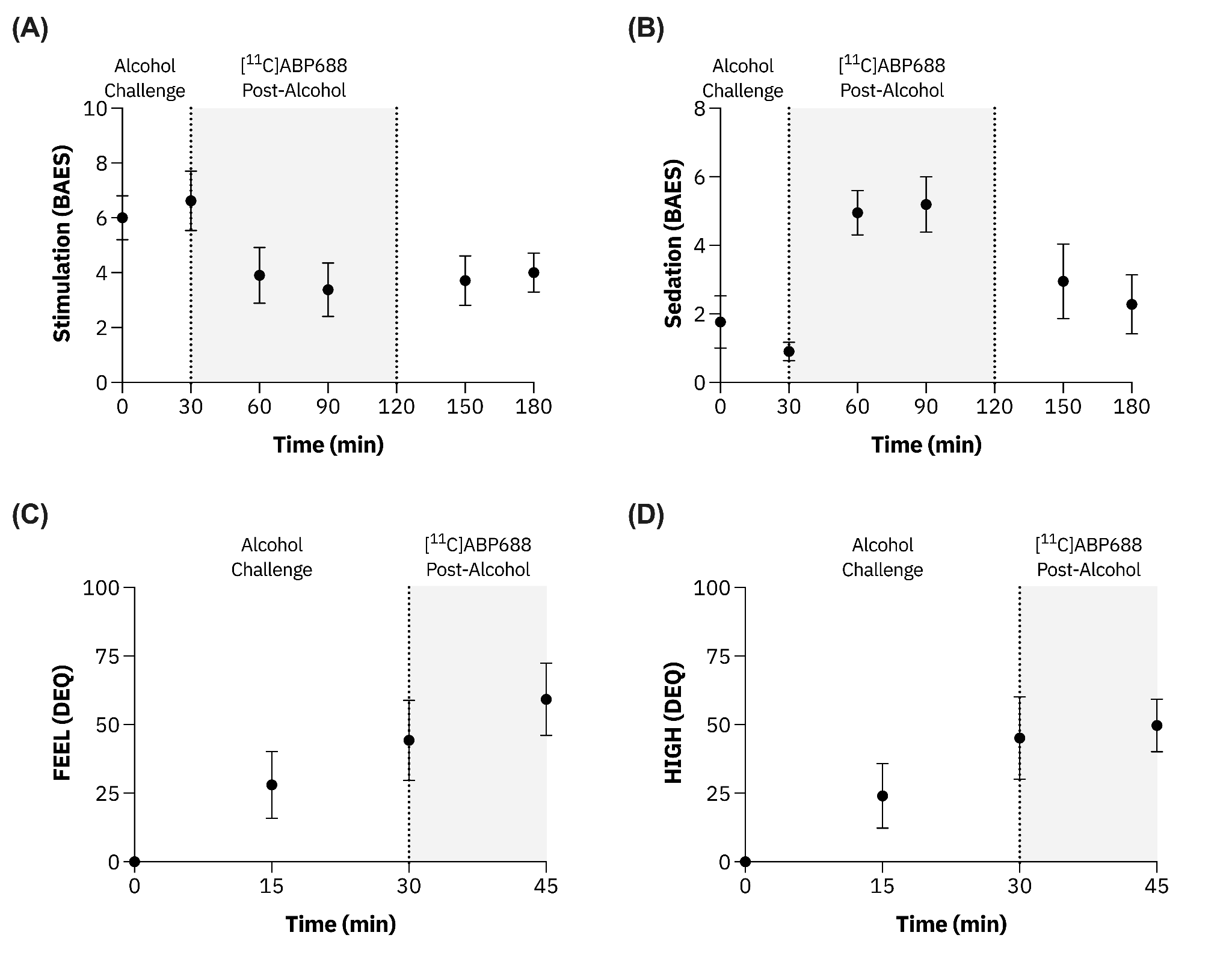


**Supp. Fig. 1.** Behavioral data showing the effects of alcohol consumption on (A) stimulation and (B) sedation, measured by the Biphasic Alcohol Effects Scale (BAES), and (C) FEEL and (D) HIGH ratings, measured by the Drug Effects Questionnaire (DEQ). Data points represent mean values, and error bars indicate the standard error of the mean (SEM). The shaded areas denote the period of [^11^C]ABP688 PET imaging post-alcohol challenge.

**Supp. Table 1.** [^11^C]ABP688 *V*_T_ before and after the alcohol challenge (n = 4)

| **Region** | **Baseline**  ***V*_T_** | **Post-alcohol**  ***V*_T_** | **Two-sample t-test**  **(p value)** |
| --- | --- | --- | --- |
| Frontal Cortex | 3.75 ± 0.69  (range: 3.05 – 4.73) | 3.77 ± 0.35  (range: 3.63 – 4.22) | 0.938 |
| Temporal Cortex | 3.78 ± 0.71  (range: 3.10 – 4.78) | 3.77 ± 0.34  (range: 3.43 – 4.24) | 0.944 |
| Striatum | 3.53 ± 0.66  (range: 2.91 – 4.47) | 3.51 ± 0.38  (range: 3.24 – 4.06) | 0.890 |
| Hippocampus | 3.46 ± 0.45  (range: 2.72 – 4.77) | 3.41 ± 0.45  (range: 3.01 – 4.06) | 0.836 |
| Cerebellum | 1.91 ± 0.35  (range: 1.66 – 2.44) | 2.03 ± 0.34  (range: 1.71 – 2.46) | 0.144 |
| *V*_T_ values were calculated using the two-tissue compartment model  All regions were grey-matter segemented using the CAT segmentation.  Uncorrected p value results are presented.  Data is reported as Mean ± Standard Deviation and range. | | | |

**Supp. Table 2.** [^11^C]ABP688 *K*_1_ before and after the alcohol challenge (n = 4)

| **Region** | **Baseline**  ***K*_1_** | **Post-alcohol**  ***K*_1_** | **Two-sample t-test**  **(p value)** |
| --- | --- | --- | --- |
| Frontal Cortex | 0.20 ± 0.03  (range: 0.16 – 0.25) | 0.22 ± 0.03  (range: 0.19 – 0.27) | 0.145 |
| Temporal Cortex | 0.17 ± 0.03  (range: 0.14 – 0.21) | 0.19 ± 0.02  (range: 0.17 – 0.22) | 0.194 |
| Striatum | 0.19 ± 0.02  (range: 0.17 – 0.21) | 0.21 ± 0.01  (range: 0.21 – 0.22) | 0.149 |
| Hippocampus | 0.13 ± 0.02  (range: 0.11 – 0.15) | 0.15 ± 0.01  (range: 0.14 – 0.16) | 0.053 |
| Cerebellum | 0.20 ± 0.01  (range: 0.19 – 0.22) | 0.20 ± 0.01  (range: 0.19 – 0.21) | 0.593 |
| *K*_1_ values were calculated using the two-tissue compartment model  All regions were grey-matter segemented using the CAT segmentation.  Uncorrected p value results are presented.  Data is reported as Mean ± Standard Deviation and range. | | | |

**Supp. Table 3.** [^11^C]ABP688 Standardized Uptake Value (SUV) values before and after the alcohol challenge (n = 7)

| **Region** | **Baseline**  **SUV** | **Post-alcohol**  **SUV** | **Two-sample t-test**  **(p value)** |
| --- | --- | --- | --- |
| Frontal Cortex | 1.05± 0.10  (range: 0.96 – 1.25) | 0.94 ± 0.23  (range: 0.70 – 1.40) | 0.089 |
| Temporal Cortex | 1.09 ± 0.20  (range: 0.96 – 1.47) | 0.96 ± 0.17  (range: 0.69 – 1.29) | 0.034 |
| Striatum | 0.89 ± 0.21  (range: 0.69 – 1.32) | 0.92 ± 0.17  (range: 0.60 – 1.18) | 0.700 |
| Hippocampus | 0.96 ± 0.20  (range: 0.62 – 1.30) | 0.89 ± 0.14  (range: 0.73 – 1.13) | 0.316 |
| Cerebellum | 0.57 ± 0.12  (range: 0.40 – 0.68) | 0.55 ± 0.11  (range: 0.37 – 0.68) | 0.549 |
| SUV values were calculated between 60-90 mins post injection.  All regions were grey-matter segemented using the CAT segmentation.  Uncorrected p value results are presented.  Data is reported as Mean ± Standard Deviation and range. | | | |

**Supp. Table 4.** Partial correlation analysis between post-alcohol Δ*BP*_ND_ values and peak blood alcohol levels (BAL), drinks in the last 30 days as measures with Alcohol Timeline Followback questionnaire, biphasic alcohol effect scale (BAES) sedation (90 min post-alcohol) and simulation (30 min post-alcohol), drug effect questionnaire (DEQ) FEEL and DEQ HIGH (30 min post-alcohol) (*n*=7).

| **Region** |  | **Peak**  **BAL** | **Drinks last 30 days** | **BAES**  **Sedation**  **(90 min)** | **BAES**  **Stimulation**  **(30 min)** | **DEQ**  **FEEL**  **(30 min)** | **DEQ**  **HIGH**  **(30 min)** |
| --- | --- | --- | --- | --- | --- | --- | --- |
| Frontal | Spearman's rho | 0.04 | -0.07 | 0.68 | 0.68 | -0.36 | -0.36 |
|  | p value | 0.939 | 0.879 | 0.094 | 0.090 | 0.432 | 0.432 |
| Temporal | Spearman's rho | 0.04 | -0.07 | 0.68 | 0.68 | -0.36 | -0.36 |
|  | p value | 0.939 | 0.879 | 0.094 | 0.090 | 0.432 | 0.432 |
| Striatum | Spearman's rho | 0.04 | -0.29 | 0.75 | 0.70 | -0.43 | -0.43 |
|  | p value | 0.939 | 0.535 | 0.052 | 0.078 | 0.337 | 0.337 |
| Hippocampus | Spearman's rho | 0.11 | -0.46 | 0.71 | 0.67 | -0.36 | -0.36 |
|  | p value | 0.819 | 0.294 | 0.071 | 0.102 | 0.432 | 0.432 |

**Supp. Table 5.** Partial correlation analysis between post-alcohol Δ*R_1_* values and peak blood alcohol levels (BAL), drinks in the last 30 days as measures with Alcohol Timeline Followback questionnaire, biphasic alcohol effect scale (BAES) sedation (90 min post-alcohol) and simulation (30 min post-alcohol), drug effect questionnaire (DEQ) FEEL and DEQ HIGH (30 min post-alcohol) (*n*=7).

| **Region** |  | **Peak**  **BAL** | **Drinks last 30 days** | **BAES**  **Sedation**  **(90 min)** | **BAES**  **Stimulation**  **(30 min)** | **DEQ**  **FEEL**  **(30 min)** | **DEQ**  **HIGH**  **(30 min)** |
| --- | --- | --- | --- | --- | --- | --- | --- |
| Frontal | Spearman's rho | 0.86 | -0.11 | -0.29 | -0.09 | 0.68 | 0.68 |
|  | p value | 0.014 | 0.819 | 0.535 | 0.848 | 0.094 | 0.094 |
| Temporal | Spearman's rho | 0.79 | -0.50 | -0.36 | -0.23 | 0.43 | 0.43 |
|  | p value | 0.036 | 0.253 | 0.432 | 0.613 | 0.337 | 0.337 |
| Striatum | Spearman's rho | 0.32 | -0.07 | 0.00 | 0.02 | -0.11 | -0.11 |
|  | p value | 0.482 | 0.879 | 1.000 | 0.969 | 0.819 | 0.819 |
| Hippocampus | Spearman's rho | 0.14 | -0.43 | 0.46 | 0.45 | -0.11 | -0.11 |
|  | p value | 0.760 | 0.337 | 0.294 | 0.310 | 0.819 | 0.819 |
